# Clinical Spectrum and Early Renal Functional Variability in a Nationwide Greek Pediatric HNF1B Multicenter Cohort

**DOI:** 10.3390/jcm15176590

**Published:** 2026-08-26

**Authors:** John Dotis, Argyroula Zampetoglou, Varvara Askiti, Maria Bitsori, Ekaterini Siomou, Chrysoula Kosmeri, Nikoleta Printza, Antonia Kondou, Athina Ververi, Charalampos Kapogiannis, Anastasios Kapogiannis, Despoina Tramma, Konstantinos Kollios, Maria Fourikou, Hellenic Society of Pediatric Nephrology (HSPN)

**Affiliations:** 1 Third Department of Pediatrics, Hippokration Hospital, Aristotle University of Thessaloniki, 54642 Thessaloniki, Greece; kkollios@auth.gr (K.K.); mfour@auth.gr (M.F.); 2 Pediatric Nephrology Department, “Panagiotis & Aglaia Kyriakou” Children’s Hospital, 11527 Athens, Greece; azampetoglou@hotmail.com (A.Z.); vaskiti@gmail.com (V.A.); 3 Department of Pediatrics, University General Hospital of Heraklion, 71500 Heraklion, Greece; bitmar2@gmail.com; 4 Department of Pediatrics, University Hospital of Ioannina, University of Ioannina, 45110 Ioannina, Greece; eksiomou@uoi.gr (E.S.); chrisa.kosmeri@gmail.com (C.K.); 5 First Department of Pediatrics, Hippokration Hospital, Aristotle University of Thessaloniki, 54642 Thessaloniki, Greece; nprintza@gmail.com (N.P.); antkontou@auth.gr (A.K.); 6 Centre of Genetics for Rare Diseases, Papageorgiou Hospital, 56403 Thessaloniki, Greece; athina.ververi@nhs.net; 7 Second Department of Pediatrics, Athens Medical Group, 14562 Athens, Greece; hariskapogiannis@gmail.com; 8 Department of Pediatrics, IASO Children’s Hospital, 15123 Athens, Greece; ankapogiannis@gmail.com; 9 Fourth Department of Pediatrics, “G. Papageorgiou” General Hospital, Aristotle University of Thessaloniki, 54124 Thessaloniki, Greece; dtramma@auth.gr

**Keywords:** HNF1B nephropathy, 17q12 microdeletion, renal cysts, CAKUT, hypomagnesemia, pediatric nephrology, genotype–phenotype correlation

## Abstract

**Background/Objectives**: Hepatocyte nuclear factor 1 beta (HNF1B)-associated disease is a major monogenic cause of pediatric cystic kidney disease. This study characterized the clinical, genetic, renal, and extrarenal spectrum of HNF1B-associated disease in a nationwide Greek pediatric cohort, with emphasis on renal functional variability during childhood. **Methods**: In this retrospective multicenter cohort study, 20 children aged <18 years with molecularly characterized HNF1B-associated disease were identified from eight pediatric nephrology centers across Greece. Clinical, genetic, laboratory, imaging, and kidney function data were analyzed. Estimated glomerular filtration rate (eGFR) was assessed at diagnosis and latest follow-up, and genotype–phenotype comparisons were performed. **Results**: Median age at diagnosis was 5.0 years. Copy-number variants were identified in 12 patients (60.0%), including ten 17q12 microdeletions and two whole-gene HNF1B deletions, while eight patients (40.0%) carried intragenic variants. Renal cysts were present in 95.0% of patients, hyperechogenic kidneys in 70.0%, and congenital anomalies of the kidney and urinary tract (CAKUT) in 20.0%. Hypomagnesemia was observed in 25.0% of patients, while hyperuricemia was identified in 29.4% of those with available measurements. Longitudinal eGFR data were available for 19 patients. Over a median follow-up of 5.0 years, mean eGFR remained largely unchanged (84.5 ± 19.8 vs. 84.0 ± 16.7 mL/min/1.73 m^2^); however, substantial interindividual variability was observed. No statistically significant genotype–phenotype associations were identified, although hypomagnesemia was more frequent among patients with deletion-type defects. **Conclusions**: This nationwide Greek pediatric cohort highlights the marked phenotypic heterogeneity of HNF1B-associated disease, characterized by predominant structural renal abnormalities and relatively stable kidney function during childhood. Larger studies are needed to better define genotype-specific outcomes.

## 1. Introduction

Hepatocyte nuclear factor 1 beta (HNF1B) is a homeodomain-containing transcription factor essential for the development and function of several organs, including the kidneys, pancreas, liver and genitourinary tract [1,2]. Pathogenic variants of the HNF1B gene on chromosome 17q12, including intragenic mutations and whole-gene deletions, cause a multisystem disorder with marked phenotypic variability [1]. Initially described in association with renal cysts and diabetes syndrome (RCAD) and maturity-onset diabetes of the young type 5 (MODY5), HNF1B-associated disease is now recognized as a major monogenic disorder of kidney and urinary tract development [1,3].

Renal involvement is the most common and often the earliest manifestation in childhood [4,5]. Renal abnormalities include congenital anomalies of the kidney and urinary tract (CAKUT), renal hypoplasia, dysplasia, multicystic dysplastic kidney, hyperechogenic kidneys and renal cysts [4,6]. HNF1B defects represent one of the most common monogenic causes of CAKUT in pediatric cohorts, making genetic testing an important component of the diagnostic evaluation of children with developmental or cystic kidney disease [5,7].

Clinical presentation is highly variable. Although many patients maintain stable kidney function, others develop chronic kidney disease (CKD) early in life, occasionally progressing to kidney failure during childhood or adolescence [4,8]. Disease severity may differ even among relatives carrying the same genetic variant, suggesting the influence of additional modifying factors [1].

Beyond structural kidney abnormalities, HNF1B-associated disease is frequently associated with tubular dysfunction and extrarenal manifestations. Hypomagnesemia and hyperuricemia are common biochemical findings and may provide useful diagnostic clues [4,7]. Extrarenal features include abnormalities of glucose metabolism, pancreatic hypoplasia, liver dysfunction, genital tract malformations and neurodevelopmental disorders [1,3]. Since these manifestations may appear progressively, long-term multidisciplinary follow-up is required.

Recent multicenter studies have improved understanding of pediatric HNF1B nephropathy and suggested genotype–phenotype associations between patients with 17q12 deletions and those with intragenic variants [4,5,8]. However, pediatric data remain limited, particularly from Southern Europe, and few studies have examined longitudinal renal function during childhood.

Therefore, this study aimed to describe the clinical, genetic, renal, tubular, and extrarenal features of pediatric HNF1B-associated nephropathy in a nationwide Greek multicenter cohort of 20 molecularly characterized patients. By specifically evaluating longitudinal kidney function trajectories and kidney outcomes during pediatric follow-up, this cohort provides insight into the early clinical course of the disease.

## 2. Materials and Methods

### 2.1. Study Design and Participating Centers

This retrospective multicenter cohort study was conducted under the collaborative framework of the Hellenic Society of Pediatric Nephrology (HSPN). The study aimed to systematically characterize the clinical, genetic, renal, and extrarenal manifestations of pediatric HNF1B-associated nephropathy in Greece.

Pediatric patients with molecularly characterized HNF1B-associated disease were retrospectively identified through participating pediatric nephrology centers across Greece. Data were collected from eight centers located in four cities (Athens, Thessaloniki, Heraklion and Ioannina), encompassing the largest pediatric nephrology referral units in the country. The participating centers included the Department of Pediatric Nephrology, “P. & A. Kyriakou” Children’s Hospital, Athens; IASO Children’s Hospital, Athens; Athens Medical Group, Athens; the First and Third Departments of Pediatrics, Aristotle University of Thessaloniki, Hippokration General Hospital, Thessaloniki; the Fourth Department of Pediatrics, Aristotle University of Thessaloniki, Papageorgiou General Hospital; the Department of Pediatrics, University Hospital of Heraklion, Heraklion; and the Department of Pediatric Nephrology, University Hospital of Ioannina, Ioannina.

Clinical and genetic information was collected using a standardized data collection form specifically designed for the study. All data were anonymized prior to analysis and underwent centralized review by the coordinating center. This cohort represents, to our knowledge, the first nationwide multicenter collaborative study of molecularly characterized pediatric HNF1B-associated nephropathy performed in Greece.

### 2.2. Patient Selection

Patients were eligible for inclusion if they met all of the following criteria: (i) age < 18 years at diagnosis or during follow-up; (ii) HNF1B-associated disease supported by molecular genetic testing, defined as either a pathogenic or likely pathogenic intragenic HNF1B variant, a chromosome 17q12 deletion encompassing the HNF1B gene, or, in selected cases, a variant of uncertain significance (VUS) accompanied by a highly compatible clinical phenotype; and (iii) availability of sufficient clinical, genetic, and renal phenotypic data for analysis.

Patients lacking molecular evidence of HNF1B-associated disease were excluded. Variants classified as benign or likely benign were not considered eligible for inclusion. In cases of VUS, inclusion was considered only when the variant was reported by the diagnostic laboratory as potentially disease-associated and when the patient exhibited a highly compatible HNF1B-associated phenotype, including characteristic renal manifestations and/or relevant extrarenal features. All 20 patients identified by the participating centers met the predefined eligibility criteria and were included in the final cohort; no eligible patient was excluded.

Only one entry per patient was included in the study. When serial evaluations were available, baseline characteristics at diagnosis and the most recent follow-up data were recorded.

### 2.3. Genetic Evaluation

The diagnosis of HNF1B-associated disease was established through molecular genetic testing performed as part of routine clinical care at participating centers. Genetic testing methodologies included next-generation sequencing (NGS)-based gene panels, whole-exome sequencing (WES), Sanger sequencing for variant confirmation when applicable, and chromosomal microarray analysis or multiplex ligation-dependent probe amplification (MLPA) for the detection of copy-number variants, including chromosome 17q12 deletions encompassing the HNF1B gene.

Genetic findings and their clinical classifications were extracted from the original molecular diagnostic reports. Sequence variants were classified according to the American College of Medical Genetics and Genomics/Association for Molecular Pathology (ACMG/AMP) criteria and copy-number variants according to the American College of Medical Genetics and Genomics/Clinical Genome Resource (ACMG/ClinGen) technical standards. Patients carrying pathogenic or likely pathogenic HNF1B-related genetic findings were eligible for inclusion. In addition, two patients carrying VUS were retained in the cohort because the variants had been reported by the diagnostic laboratories as potentially disease-associated and the patients exhibited highly compatible HNF1B-associated phenotypes, according to the predefined study eligibility criteria. A patient-level summary of the clinical context supporting cohort inclusion of the two VUS cases is provided in Supplementary Table S1. These variants remain classified as VUS and were not reclassified as pathogenic on the basis of phenotype alone.

Detected abnormalities were categorized as intragenic HNF1B variants, whole-gene HNF1B deletions, or chromosome 17q12 microdeletions encompassing the HNF1B locus. Detailed genotype distributions and variant characteristics are presented in the Results section.

### 2.4. Data Collection

Clinical, genetic, laboratory, and imaging data were retrospectively collected from medical records using a standardized study form. Recorded variables included demographic characteristics (sex, year of birth, age at diagnosis, and family history), genetic findings (indication for testing, testing method, variant type, and inheritance pattern), renal manifestations (renal cysts, congenital anomalies of the kidney and urinary tract [CAKUT], hyperechogenic kidneys, renal dysplasia/hypoplasia, and kidney function), tubular abnormalities (hypomagnesemia and hyperuricemia), and extrarenal manifestations, including diabetes mellitus/MODY, pancreatic abnormalities, liver involvement, genital tract malformations, and other reported extrarenal features. Longitudinal data included estimated glomerular filtration rate (eGFR) at diagnosis, most recent available eGFR, and duration of follow-up. For genotype–phenotype analyses, genetic defects were categorized as deletion-type defects (17q12 microdeletions or whole-gene HNF1B deletions) and intragenic HNF1B variants. The standardized form recorded whether hypomagnesemia and hyperuricemia were present according to age-adjusted local reference ranges; the corresponding numerical serum magnesium and uric acid values were not collected in the centralized dataset.

### 2.5. Kidney Function Assessment

Kidney function was assessed using estimated glomerular filtration rate (eGFR), calculated using the updated bedside Schwartz equation [9]. eGFR values at diagnosis and at the most recent follow-up visit were collected from the medical records of participating centers. For patients with longitudinal follow-up data, changes in kidney function over time were evaluated by comparing baseline and latest available eGFR measurements.

Annual eGFR change was calculated as the difference between follow-up and baseline eGFR divided by the duration of follow-up (mL/min/1.73 m^2^/year). Given the known heterogeneity of HNF1B-associated nephropathy, particular emphasis was placed on the assessment of renal functional variability during childhood follow-up. The proportion of patients with preserved kidney function and those exhibiting reduced eGFR at diagnosis and follow-up was also evaluated descriptively. Longitudinal analyses were restricted to patients with both baseline and follow-up eGFR measurements and a calculable follow-up interval.

### 2.6. Definitions

For the purposes of this study, renal cysts were defined as one or more cystic renal lesions detected by ultrasonography, while CAKUT was defined as any structural abnormality of the kidneys and/or urinary tract identified on prenatal or postnatal imaging. Hypomagnesemia and hyperuricemia were defined as serum magnesium and uric acid concentrations outside the age-adjusted reference ranges of the respective participating centers. Extrarenal manifestations were systematically assessed and recorded when documented in the medical records, including pancreatic, hepatic, genital, endocrine, metabolic, and neurodevelopmental abnormalities. Finally, family history was considered positive when HNF1B-associated disease, renal cystic disease, CAKUT, diabetes mellitus, or other compatible manifestations were present in a first- or second-degree relative.

### 2.7. Ethics

The study was conducted in accordance with the ethical principles of the Declaration of Helsinki. This was a retrospective, multicenter observational study based exclusively on data obtained during routine clinical care. Clinical, laboratory, imaging, and genetic information were retrospectively collected from the medical records of participating centers and anonymized prior to inclusion in the study database and data analysis. No interventions were performed for research purposes, and no identifiable personal information was collected, stored, or reported.

Given the retrospective and non-interventional nature of the study, the exclusive use of fully anonymized data, and the absence of any procedures performed specifically for research purposes, formal ethical review and approval were not required according to institutional policies governing retrospective observational studies. Individual informed consent was waived because the use of fully anonymized data precluded identification of individual patients.

### 2.8. Statistical Analysis

Data from all participating centers were entered into a standardized Microsoft Excel for Microsoft 365 (Microsoft Corporation, Redmond, WA, USA) database and subsequently analyzed using GraphPad InStat version 3.10 (GraphPad Software Inc., San Diego, CA, USA). Continuous variables were tested for normality and are presented as mean ± standard deviation (SD) or median and interquartile range (IQR), as appropriate. Categorical variables are presented as frequencies and percentages. Renal functional evolution during follow-up was assessed descriptively by comparing eGFR values at diagnosis and at the most recent available follow-up evaluation. Annual eGFR change was additionally calculated for patients with available longitudinal data.

Given the descriptive nature of the study, the rarity of HNF1B-associated disease, and the limited sample size, the statistical analyses were primarily exploratory and should be interpreted with caution. Due to the limited sample size and low event counts, multivariable analyses were not performed. Exploratory comparisons between genotype groups (deletion-type defects versus intragenic variants) were conducted using Fisher’s exact test and Mann–Whitney U test, as appropriate. Statistical significance was defined as a two-sided *p*-value < 0.05. A sensitivity analysis excluding the two patients carrying VUS was performed. Potential co-occurrence patterns among abnormalities were examined descriptively; formal pairwise testing was not performed when event counts were too sparse to yield stable estimates. For categorical genotype comparisons, effect sizes were expressed as percentage-point differences (deletion minus intragenic group). Rank-biserial correlation was calculated as the effect size for the Mann–Whitney U comparison of annual eGFR change.

ChatGPT (GPT-5.6 Sol; OpenAI, San Francisco, CA, USA) was used exclusively to support the design and visual preparation of the figures. All scientific content, data selection, interpretation, and final visual presentation were critically reviewed, edited, and approved by the authors. No AI tool was used for data generation or statistical analysis.

## 3. Results

### 3.1. Study Population and Participating Centers

**Figure 1:**
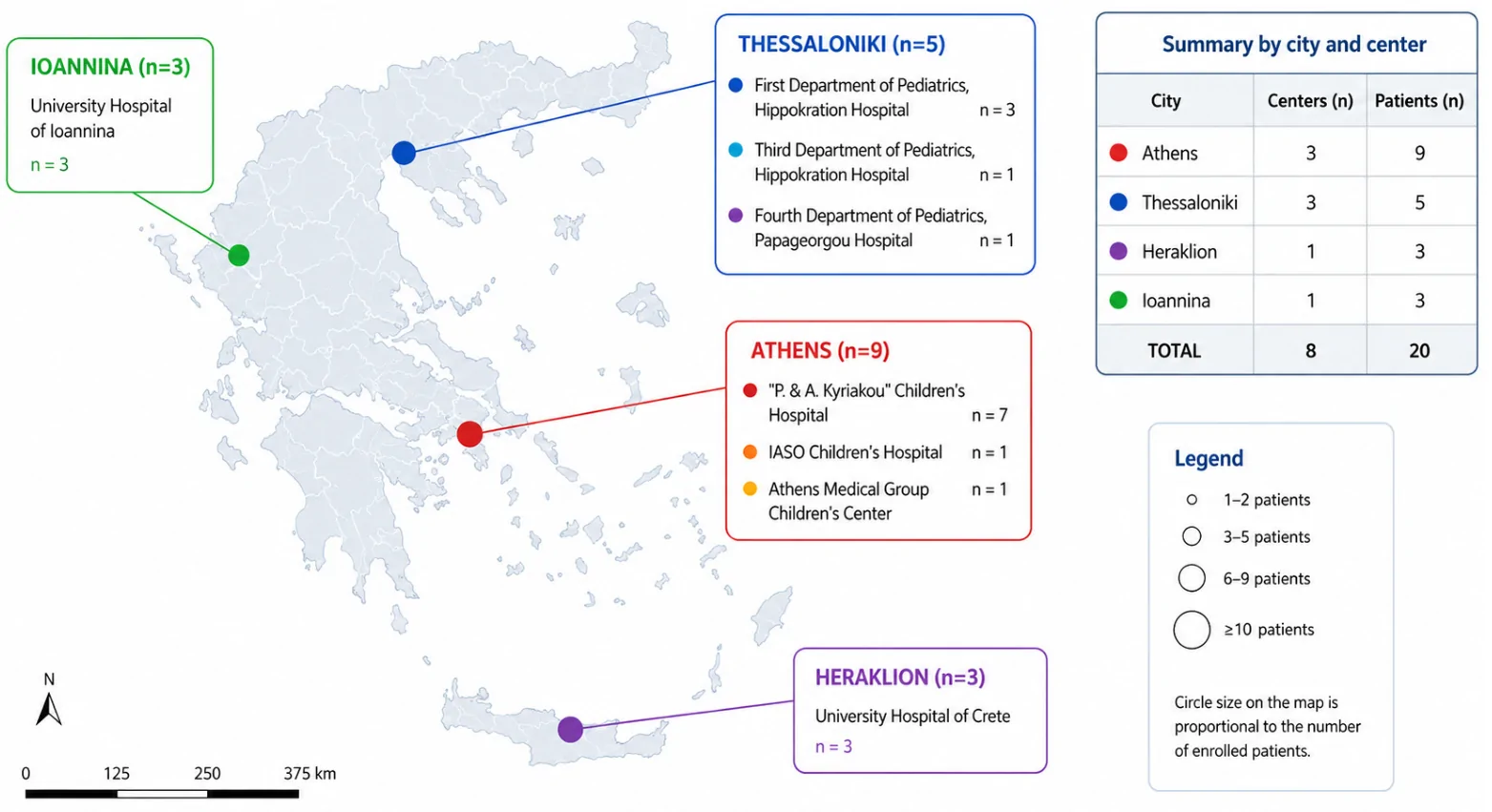
Geographic distribution of the Greek multicenter pediatric HNF1B cohort.

A total of 20 children aged <18 years with molecularly characterized HNF1B-associated disease were enrolled from eight pediatric nephrology centers across four Greek cities (Figure 1). Participating institutions included three centers in Athens, three in Thessaloniki, and one center each in Heraklion and Ioannina, reflecting the multicenter nationwide design of the study.

Twenty children with molecularly characterized HNF1B-associated nephropathy were identified across eight pediatric nephrology centers located in four cities in Greece. Athens contributed nine patients from three centers (General Children’s Hospital “P. & A. Kyriakou”, IASO Children’s Hospital, and Athens Medical Group), Thessaloniki contributed five patients from three centers (First Department of Pediatrics, Hippokration Hospital; Third Department of Pediatrics, Hippokration Hospital; and Fourth Department of Pediatrics, Papageorgiou Hospital), while Heraklion and Ioannina each contributed three patients from one tertiary referral center. Circle size is proportional to the number of enrolled patients.

The demographic characteristics of the cohort are summarized in Table 1. The study population showed a male predominance, and diagnoses were established throughout childhood, with a median age at diagnosis of 5 years. A positive family history suggestive of inherited disease was documented in approximately one-third of patients, whereas most cases occurred in the absence of a reported familial history.

### 3.2. Genetic Findings

Detailed genetic findings are summarized in Table 2. Among the 20 children with molecularly characterized HNF1B-associated disease, 12 (60.0%) carried copy-number variants (CNVs), including ten recurrent 17q12 microdeletions and two whole-gene HNF1B deletions. The remaining eight patients (40.0%) harbored intragenic HNF1B variants, comprising three frameshift, two nonsense, and three missense variants. Recurrent 17q12 microdeletions represented the most common genetic abnormality, accounting for half of the cohort (50.0%). Most identified genetic alterations were classified as pathogenic or likely pathogenic, whereas two missense variants remained classified as variants of uncertain significance (VUS).

### 3.3. Renal Phenotype

The renal phenotype of the cohort is summarized in Table 3. Renal cysts represented the predominant imaging finding and were identified in nearly all patients. Additional structural abnormalities included congenital anomalies of the kidney and urinary tract (CAKUT), while increased renal echogenicity was observed in a substantial proportion of cases, highlighting the broad spectrum of renal involvement associated with HNF1B defects.

Reduced kidney function was frequently present at diagnosis, with 60.0% of patients demonstrating an eGFR below 90 mL/min/1.73 m^2^. The same proportion remained below this threshold at the most recent follow-up evaluation. Nevertheless, most children were classified within CKD stages G1–G2 at the most recent follow-up, whereas only a small minority had reached CKD stage G3 or higher. Renal functional trajectories exhibited substantial interindividual variability, ranging from stable kidney function over time to persistent reductions in eGFR.

Paired baseline and follow-up eGFR measurements were available for 19 of the 20 patients. Mean baseline eGFR was 84.5 ± 19.8 mL/min/1.73 m^2^, compared with 84.0 ± 16.7 mL/min/1.73 m^2^ at the most recent follow-up. Mean eGFR therefore remained relatively stable, although substantial interindividual variability was observed.

### 3.4. Tubular and Extrarenal Manifestations

The frequency and distribution of tubular and extrarenal manifestations are presented in Table 4.

Hyperuricemia was identified in 5 of 17 patients with available measurements (29.4%), while hypomagnesemia was present in 5 of 20 patients (25.0%). Pancreatic involvement was observed in 4 children (20.0%), while neurodevelopmental disorders were documented in 3 (15.0%). Diabetes mellitus/MODY5, genital tract anomalies, and gastrointestinal abnormalities were each present in 2 patients (10.0%). Cardiac involvement was uncommon and was recorded in a single patient (5.0%). These findings further illustrate the multisystem nature and phenotypic heterogeneity of HNF1B-associated disease. At the individual level, the cardiac abnormality was documented in Patient 3 (c.832G>A, p.Val278Met; VUS), who also had short stature. The two genital anomalies included in the final cohort analysis occurred in Patients 15 and 20, both of whom carried 17q12 microdeletions. The available centralized records did not provide sufficiently uniform anatomical detail to further subtype these abnormalities. Both patients with diabetes/MODY5 also had pancreatic abnormalities, whereas neurodevelopmental and genital abnormalities were confined to the deletion group. No other recurrent combination involved enough patients to support a reliable variant-specific analysis.

### 3.5. Genotype–Phenotype Analysis

Genotype–phenotype comparisons between patients carrying deletion-type defects (17q12 microdeletions or whole-gene HNF1B deletions) and those harboring intragenic variants are presented in Table 5.

No statistically significant differences were detected between the two groups with respect to the evaluated renal, tubular, and extrarenal manifestations. However, hypomagnesemia was numerically more frequent among patients with deletion-type defects (41.7% vs. 0%, *p* = 0.055). Exclusion of the two patients carrying VUS did not materially alter the descriptive findings or the exploratory genotype–phenotype comparisons. The largest percentage-point differences were observed for hypomagnesemia (+41.7), hyperuricemia (−31.8 percentage points among patients with available measurements), and diabetes/MODY5, neurodevelopmental disorders, and reduced eGFR at last follow-up (differences of 25.0 percentage points in magnitude).

Although no significant genotype–phenotype associations were identified, some descriptive differences between genotype groups were observed. Diabetes mellitus/MODY5 was present only among patients with intragenic variants, whereas neurodevelopmental disorders were observed exclusively in the deletion group. In addition, reduced eGFR at the most recent follow-up appeared more common among patients with intragenic variants, although this difference did not reach statistical significance. Renal structural abnormalities showed a broadly similar distribution across genotype categories.

### 3.6. Longitudinal Kidney Function by Genotype

Among patients with longitudinal data, follow-up duration was similar between genotype groups, with a median follow-up of 5.0 years (IQR 2.0–5.5) among 11 patients with deletion-type defects and 4.5 years (IQR 1.9–7.0) among 8 patients carrying intragenic variants.

**Figure 2:**
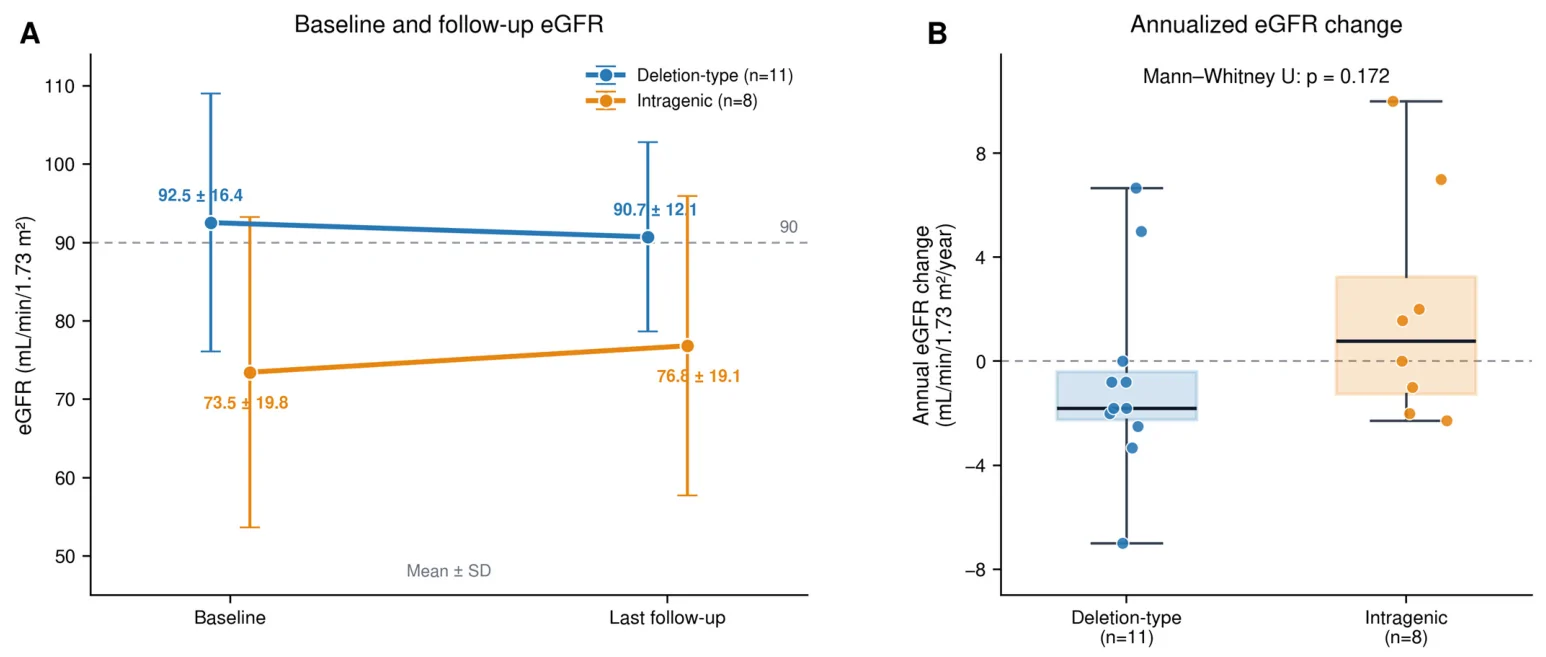
Longitudinal kidney function according to HNF1B genotype category. (**A**) Mean estimated glomerular filtration rate (eGFR) at diagnosis and last follow-up (mean ± SD) in patients with deletion-type defects (*n* = 11) and intragenic variants (*n* = 8). The dashed horizontal line marks 90 mL/min/1.73 m^2^. (**B**) Individual annualized eGFR change by genotype category; boxes show the interquartile range, horizontal lines the median, whiskers the range, and points individual patients. The dashed horizontal line marks zero annual change. Longitudinal data were available for 19 patients. The between-group comparison was not statistically significant (Mann–Whitney U test, *p* = 0.172).

Mean baseline eGFR was 92.5 ± 16.4 mL/min/1.73 m^2^ in the deletion group and 73.5 ± 19.8 mL/min/1.73 m^2^ in the intragenic group; the corresponding values at last follow-up were 90.7 ± 12.1 and 76.8 ± 19.1 mL/min/1.73 m^2^, respectively (Figure 2A). Patients with deletion-type defects showed a mean annual eGFR change of −0.76 ± 3.76 mL/min/1.73 m^2^/year, compared with +1.91 ± 4.42 mL/min/1.73 m^2^/year among those carrying intragenic variants. Despite this numerical difference, annual eGFR change did not differ significantly between genotype groups (Mann–Whitney U test, *p* = 0.172) (Figure 2B). The rank-biserial correlation (deletion versus intragenic group) was −0.39, indicating lower annual eGFR change in the deletion group, although the estimate remained statistically imprecise in this small exploratory cohort.

## 4. Discussion

In this nationwide multicenter study of children with molecularly characterized HNF1B-associated disease, we observed substantial phenotypic heterogeneity despite a shared molecular diagnosis. Although modest in absolute numbers, the present cohort likely captures a substantial proportion of children with molecularly characterized HNF1B-associated disease currently followed in Greece, thereby providing a representative overview of the pediatric spectrum of the disorder at a national level. The presence of a positive family history in 35% of patients further illustrates the variable penetrance and marked phenotypic heterogeneity that characterize HNF1B-associated disease. Structural renal abnormalities, particularly renal cysts and hyperechogenic kidneys, represented the predominant clinical manifestations, while extrarenal involvement was also common. Despite the broad spectrum of renal and extrarenal features, kidney function remained largely preserved during childhood, with overall stable eGFR values during a median follow-up of approximately five years. In addition, genotype-stratified analyses suggested several clinically relevant trends, including a higher frequency of hypomagnesemia and neurodevelopmental abnormalities among patients with deletion-type defects. These findings are consistent with the recognized multisystem nature of HNF1B-associated disease and further highlight the marked clinical variability that characterizes this disorder [1,4,5,8].

Structural renal abnormalities constituted the dominant clinical phenotype in our cohort, with renal cysts identified in 95% of patients, hyperechogenic kidneys in 70%, and CAKUT in 20%. These findings are consistent with the central role of HNF1B in kidney development, as disruption of this transcription factor results in abnormalities of nephrogenesis and urinary tract development [1,2]. The predominance of cystic and developmental renal abnormalities observed in our patients is in agreement with previous European and Asian cohorts, in which renal cysts and other structural kidney abnormalities represented the hallmark manifestations of HNF1B-associated disease [5,8,10]. Similar findings have also been reported in a recent Greek single-center study highlighting the phenotypic variability associated with HNF1B defects [11]. Given the broad differential diagnosis of pediatric cystic kidney disease and CAKUT, HNF1B-associated disease should be considered in children presenting with renal cysts, hyperechogenic kidneys, or otherwise unexplained congenital renal anomalies [6].

Despite the high prevalence of structural renal abnormalities, kidney function remained largely stable throughout childhood in most patients. This observation is consistent with previous studies indicating that HNF1B-associated nephropathy generally follows a slowly progressive course during childhood, with preservation of renal function in the majority of affected individuals despite extensive developmental kidney abnormalities [4,8,11]. These findings are in line with previous pediatric studies demonstrating that the clinical course of HNF1B-associated nephropathy is characterized by substantial variability in renal outcomes despite broadly similar structural renal phenotypes [4,5,10]. Notably, individual eGFR trajectories were highly heterogeneous, ranging from stable renal function to gradual decline and, occasionally, apparent improvement over time. Positive annual eGFR slopes should be interpreted with caution, as they may reflect biological variability of creatinine-based eGFR estimation during growth rather than genuine improvement in kidney function [8,12]. Overall, our findings support the concept that structural kidney abnormalities and renal functional decline do not necessarily progress in parallel during childhood.

Extrarenal manifestations were common in our cohort, reinforcing the concept that HNF1B-associated disease should be viewed as a multisystem developmental disorder rather than an isolated nephropathy. Beyond renal involvement, affected individuals may develop metabolic, endocrine, pancreatic, genital, and neurodevelopmental abnormalities, reflecting the broad biological role of HNF1B across multiple organs [1,10,13]. Hypomagnesemia has emerged as one of the most characteristic metabolic manifestations of HNF1B-associated disease and may represent an important diagnostic clue even in patients with relatively preserved kidney function [14,15]. In our cohort, hypomagnesemia, hyperuricemia, pancreatic abnormalities, diabetes/MODY5, genital anomalies, and neurodevelopmental findings were observed, consistent with the marked phenotypic variability reported in previous studies [11,16]. Recent evidence further suggests that pancreatic dysfunction, diabetes, and neurodevelopmental manifestations may emerge progressively over time, highlighting the importance of multidisciplinary long-term follow-up extending beyond renal surveillance alone [3,17,18,19].

No statistically significant genotype–phenotype associations were identified in our cohort. Nevertheless, several biologically plausible genotype-specific patterns emerged, particularly regarding hypomagnesemia, neurodevelopmental abnormalities, and diabetes/MODY5.

A trend toward a higher prevalence of hypomagnesemia was observed among patients with deletion-type defects. Although not statistically significant, this finding is consistent with previous studies identifying hypomagnesemia as a characteristic manifestation of HNF1B-associated disease and suggesting increased susceptibility to magnesium wasting among individuals carrying 17q12 deletions [8,14,15,20]. These observations support the presence of a more pronounced tubular phenotype in patients with larger genomic defects.

Neurodevelopmental abnormalities were observed exclusively among patients with deletion-type defects, in agreement with the well-recognized phenotype of 17q12 microdeletion syndrome [16,19]. This finding likely reflects the loss of neighboring genes within the deleted chromosomal region rather than isolated HNF1B haploinsufficiency, emphasizing the importance of neurodevelopmental surveillance in affected children [5,8].

Conversely, diabetes/MODY5 was observed only among patients with intragenic variants. However, given the age-dependent penetrance of HNF1B-associated diabetes and the relatively young age of our cohort, this observation should be interpreted cautiously and confirmed through longer follow-up [1,17,18]. Although several genotype-specific patterns were observed, these findings should be interpreted with caution given the limited sample size and exploratory nature of the analyses. Therefore, the observed patterns should be considered hypothesis-generating rather than evidence of definitive genotype–phenotype associations.

The findings of the present study have important clinical implications. Given the marked phenotypic heterogeneity of HNF1B-associated disease, clinicians should maintain a high index of suspicion in children presenting with renal cysts, hyperechogenic kidneys, CAKUT, unexplained CKD, or characteristic extrarenal manifestations such as hypomagnesemia, pancreatic abnormalities, or diabetes [1,10,12]. A positive family history may further increase diagnostic suspicion, although de novo variants are common. In this context, genetic testing plays a pivotal role not only in establishing the diagnosis but also in guiding long-term surveillance and family counseling [20,21]. Furthermore, the broad spectrum of renal and extrarenal manifestations observed in our cohort supports a multidisciplinary follow-up strategy involving pediatric nephrologists, endocrinologists, geneticists, and other specialists as required [8,11].

Future longitudinal studies should extend beyond creatinine-based eGFR and categorical electrolyte abnormalities. Serial assessment of fractional magnesium excretion and other measures of tubular handling, together with candidate urinary markers of tubular health, injury, and inflammation such as epidermal growth factor, alpha-1 microglobulin, kidney injury molecule-1, and monocyte chemoattractant protein-1, may help identify subclinical tubular dysfunction and improve prognostic stratification; however, none has yet been validated as an HNF1B-specific prognostic biomarker [21,22]. Prospective multicenter registries using harmonized phenotyping, standardized biospecimen collection, periodic genomic reinterpretation, and follow-up through the transition to adult care could provide the sample size and longitudinal depth required to develop and externally validate genotype-informed prognostic models.

This study has several important strengths. To our knowledge, it represents the first nationwide multicenter characterization of pediatric HNF1B-associated disease in Greece, incorporating patients from eight tertiary centers across four cities. Although modest in absolute numbers, the cohort likely captures a substantial proportion of children with molecularly characterized HNF1B-associated disease currently followed in the country. Additional strengths include comprehensive molecular characterization of all included patients, a standardized review of clinical and genetic data, and longitudinal follow-up allowing assessment of early renal functional trajectories. Furthermore, the present study adds to the limited body of data on pediatric HNF1B-associated disease from Southern Europe.

Several limitations should also be acknowledged. First, the retrospective design resulted in occasional missing data, including incomplete biochemical assessment in a small number of patients. In particular, serum uric acid measurements were not uniformly available across participating centers. In addition, because the standardized study form captured these biochemical findings categorically, the numerical serum magnesium and uric acid concentrations and their ranges could not be analyzed centrally. Second, despite its national scope, the relatively small sample size limited statistical power and precluded more detailed genotype–phenotype analyses. Third, comprehensive tubular phenotyping was not uniformly available across participating centers, potentially leading to underrecognition of subtle tubular abnormalities. In addition, two patients carried variants of uncertain significance; however, both exhibited highly compatible clinical phenotypes and were therefore retained in the cohort. Finally, follow-up was restricted to childhood and adolescence, preventing assessment of adult-onset manifestations such as diabetes, progressive CKD, and other age-dependent complications. Nevertheless, the multicenter design, nationwide recruitment, and longitudinal clinical characterization provide a representative overview of the pediatric spectrum of HNF1B-associated disease in Greece.

## 5. Conclusions

The present nationwide multicenter cohort highlights the marked phenotypic heterogeneity of pediatric HNF1B-associated disease, characterized by a predominance of structural renal abnormalities and relatively stable kidney function throughout childhood. Although no statistically significant genotype–phenotype associations were identified, several biologically plausible genotype-specific patterns emerged, particularly regarding hypomagnesemia, neurodevelopmental abnormalities, and diabetes/MODY5. These findings emphasize the importance of comprehensive clinical assessment and long-term multidisciplinary follow-up in affected children. Early genetic diagnosis in children with cystic kidney disease may support appropriate clinical surveillance, facilitate genetic counseling, and enable family screening. International collaborative registries and longitudinal studies extending into adulthood will be essential to better define genotype-specific renal and extrarenal outcomes and improve risk stratification in HNF1B-associated disease.

## Figures and Tables

**Table 1:** Demographic characteristics of the study population.

Variable	Value
Male sex, *n* (%)	14 (70.0)
Female sex, *n* (%)	6 (30.0)
Age at diagnosis, years, median (IQR)	5.0 (2.0–8.0)
Family history positive, *n* (%)	7 (35.0)
Family history negative, *n* (%)	12 (60.0)
Family history unknown, *n* (%)	1 (5.0)
Participating centers, *n*	8
Cities represented, *n*	4

**Table 2:** Genetic characteristics of the Greek pediatric HNF1B cohort.

No	Center	Genetic Finding	HGVS Nomenclature	Variant Type	Classification
1	Athens A	HNF1B variant	c.176_177insA, p.Pro60Alafs*28	Frameshift	Pathogenic
2	Athens A	HNF1B variant	c.850C>T, p.Arg284*	Nonsense	Pathogenic
3	Athens A	HNF1B variant	c.832G>A, p.Val278Met	Missense	VUS
4	Athens A	Whole HNF1B deletion	Entire gene deletion	CNV	Pathogenic
5	Athens A	Whole HNF1B deletion	Entire gene deletion	CNV	Pathogenic
6	Athens A	17q12 microdeletion	chr17:34,495,978–35,812,000*	CNV—17q12 deletion	Pathogenic
7	Athens A	HNF1B variant	c.443dup, p.His149Alafs*73	Frameshift	Likely pathogenic
8	Athens B	HNF1B variant	c.529C>T, p.Arg177*	Nonsense	Pathogenic
9	Athens C	17q12 microdeletion	chr17:34,842,544–36,104,875	CNV—17q12 deletion	Pathogenic
10	Heraklion	HNF1B variant	c.866A>C, p.Asn289Thr	Missense	Likely pathogenic
11	Heraklion	17q12 microdeletion	chr17:36,486,450–37,745,134	CNV—17q12 deletion	Pathogenic
12	Heraklion	HNF1B variant	c.1207A>T, p.Ile403Phe	Missense	VUS
13	Ioannina	17q12 microdeletion	NC_000017.10:g.(?34842544)(36104875_?)del	CNV—17q12 deletion	Pathogenic
14	Ioannina	17q12 microdeletion	NC_000017.10:g.(?34842544)(36104875_?)del	CNV—17q12 deletion	Pathogenic
15	Ioannina	17q12 microdeletion	NC_000017.10:g.(?34842544)(36104875_?)del	CNV—17q12 deletion	Pathogenic
16	Thessaloniki A	HNF1B variant	c.907del, p.Arg303Alafs*24	Frameshift	Likely pathogenic
17	Thessaloniki A	17q12 microdeletion	chr17:34,495,978–36,104,886	CNV—17q12 deletion	Pathogenic
18	Thessaloniki A	17q12 microdeletion	chr17:34,495,978–36,288,312	CNV—17q12 deletion	Pathogenic
19	Thessaloniki C	17q12 microdeletion	chr17:34,842,534–36,104,886	CNV—17q12 deletion	Pathogenic
20	Thessaloniki D	17q12 microdeletion	chr17:34,842,466–36,104,935	CNV—17q12 deletion	Pathogenic

Sequence variants were classified according to ACMG/AMP criteria and copy-number variants according to ACMG/ClinGen technical standards, based on the respective diagnostic reports. * Approximate coordinates reported by array-CGH. ACMG, American College of Medical Genetics and Genomics; AMP, Association for Molecular Pathology; CNV, copy-number variant; HGVS, Human Genome Variation Society; HNF1B, hepatocyte nuclear factor 1 beta; VUS, variant of uncertain significance.

**Table 3:** Renal characteristics of the study population.

Variable	*n* (%)
Renal cysts	19 (95.0)
CAKUT	4 (20.0)
Hyperechogenic kidneys	14 (70.0)
Reduced eGFR (<90 mL/min/1.73 m^2^) at diagnosis	12 (60.0)
Reduced eGFR (<90 mL/min/1.73 m^2^) at last follow-up	12 (60.0)
CKD stage G1 (eGFR ≥90) at last follow-up	8 (40.0)
CKD stage G2 (eGFR 60–89) at last follow-up	10 (50.0)
CKD stage ≥G3 (eGFR <60) at last follow-up	2 (10.0)

CAKUT, congenital anomalies of the kidney and urinary tract; CKD, chronic kidney disease; eGFR, estimated glomerular filtration rate. CKD stages were classified according to Kidney Disease: Improving Global Outcomes (KDIGO) guidelines.

**Table 4:** Tubular and extrarenal manifestations in the study population.

Manifestation	*n* (%)
Hyperuricemia *	5 (29.4)
Hypomagnesemia	5 (25.0)
Diabetes/MODY5	2 (10.0)
Pancreatic abnormalities	4 (20.0)
Neurodevelopmental disorders	3 (15.0)
Genital anomalies	2 (10.0)
Cardiac anomalies	1 (5.0)
Gastrointestinal disorders	2 (10.0)

* Serum uric acid measurements were available for 17 of 20 patients. Numerical serum magnesium and uric acid concentrations and ranges were not available in the centralized dataset. MODY5, maturity-onset diabetes of the young type 5.

**Table 5:** Genotype–phenotype comparison.

Variable	Deletion Group (*n* = 12)	Intragenic Variants (*n* = 8)	*p* Value	Difference (Percentage Points) †
Renal cysts, *n* (%)	12 (100.0)	7 (87.5)	0.400	+12.5
CAKUT, *n* (%)	3 (25.0)	1 (12.5)	0.619	+12.5
Hyperechogenic kidneys, *n* (%)	9 (75.0)	5 (62.5)	0.642	+12.5
Hypomagnesemia, *n* (%)	5 (41.7)	0 (0.0)	0.055	+41.7
Hyperuricemia, *n*/*N* available (%) *	2/11 (18.2)	3/6 (50.0)	0.280	−31.8
Diabetes/MODY5, *n* (%)	0 (0.0)	2 (25.0)	0.147	−25.0
Pancreatic abnormalities, *n* (%)	2 (16.7)	2 (25.0)	1.000	−8.3
Neurodevelopmental disorders, *n* (%)	3 (25.0)	0 (0.0)	0.242	+25.0
Genital anomalies, *n* (%)	2 (16.7)	0 (0.0)	0.495	+16.7
Reduced eGFR at diagnosis (<90 mL/min/1.73 m^2^), *n* (%)	7 (58.3)	5 (62.5)	1.000	−4.2
Reduced eGFR at last follow-up (<90 mL/min/1.73 m^2^), *n* (%)	6 (50.0)	6 (75.0)	0.373	−25.0

The deletion group included patients carrying 17q12 microdeletions or whole-gene HNF1B deletions. *p* values were calculated using Fisher’s exact test; * Hyperuricemia data were available for 11 of 12 patients in the deletion group and 6 of 8 patients in the intragenic variant group; CAKUT, congenital anomalies of the kidney and urinary tract; eGFR, estimated glomerular filtration rate; MODY5, maturity-onset diabetes of the young type 5; † Difference was calculated as the percentage in the deletion group minus that in the intragenic variant group.

## Data Availability

The original contributions presented in this study are included in the article. Further inquiries can be directed at the corresponding author.

## Supplementary Materials

The following supporting information can be downloaded at: https://www.mdpi.com/article/10.3390/jcm15176590/s1, Table S1, Clinical context and rationale for inclusion of the two patients carrying HNF1B variants of uncertain significance.

## Abbreviations

The following abbreviations are used in this manuscript:

HNF1B	Hepatocyte Nuclear Factor 1-Beta
CAKUT	Congenital Anomalies of the Kidney and Urinary Tract
CKD	Chronic Kidney Disease
CNV	Copy-Number Variant
eGFR	Estimated Glomerular Filtration Rate
HSPN	Hellenic Society of Pediatric Nephrology
MLPA	Multiplex Ligation-Dependent Probe Amplification
MODY5	Maturity-Onset Diabetes of the Young Type 5
NGS	Next-Generation Sequencing
VUS	Variant of Uncertain Significance
WES	Whole-Exome Sequencing
