# Supplementary material for: Clinical Spectrum and Early Renal Functional Variability in a Nationwide Greek Pediatric HNF1B Multicenter Cohort
Source: J Clin Med. 2026 Aug 26;15(17):6590. doi: 10.3390/jcm15176590 (PMC13566293; doi:10.3390/jcm15176590)
Supplement: Supplementary file 1 [file jcm-15-06590-s001.zip › jcm-4475117-supplementary.pdf]

Supplementary Table S1. Clinical context and rationale for inclusion of the two patients carrying HNF1B variants of uncertain significance

Patient	HNF1B variant	Laboratory-reported classification	Inheritance	Phenotypic context relevant to cohort inclusion	Interpretation for this study
3	c.832G>A, p.Val278Met	VUS	Inherited	CAKUT with additional extrarenal findings (short stature and a cardiac abnormality); renal cysts were absent	Retained under the prespecified eligibility criteria because the diagnostic laboratory considered the variant potentially disease-associated and the phenotype was compatible with HNF1B-associated disease; not reclassified as pathogenic.
12	c.1207A>T, p.Ile403Phe	VUS	Unknown	Renal cystic disease with hyperuricemia; obesity and inflammatory bowel disease were also recorded	Retained under the prespecified eligibility criteria because the diagnostic laboratory considered the variant potentially disease-associated and the renal phenotype was compatible with HNF1B-associated disease; not reclassified as pathogenic.

Clinical compatibility was used only to support inclusion under the study's prespecified criteria and does not constitute variant reclassification. Both variants remain VUS. A sensitivity analysis excluding these two patients did not materially alter the descriptive findings or exploratory genotype–phenotype comparisons. ACMG/AMP, American College of Medical Genetics and Genomics/Association for Molecular Pathology; CAKUT, congenital anomalies of the kidney and urinary tract; VUS, variant of uncertain significance.
